# Efficacy and Safety of Poly-l-Lactic Acid in Facial Aesthetics: A Systematic Review

**DOI:** 10.3390/polym16182564

**Published:** 2024-09-11

**Authors:** Roberta Signori, Antony de Paula Barbosa, Fernando Cezar-dos-Santos, Ana Claudia Carbone, Silvio Ventura, Bryanne Brissian de Souza Nobre, Maria Luiza Boechat Borges Neves, Mariana Barbosa Câmara-Souza, Rodrigo Lorenzi Poluha, Giancarlo De la Torre Canales

**Affiliations:** 1 Egas Moniz Center for Interdisciplinary Research (CiiEM), Egas Moniz School of Health & Science, 2829-511 Caparica, Almada, Portugal; robertasignori@gmail.com; 2 Department of Research & Development, Health & Aesthetics, Antony Barbosa Institute, Belo Horizonte 31270-901, Minas Gerais, Brazil; drantonybarbosa@gmail.com; 3 Instituto Joana Borghetti, Foz do Iguaçu 85851-220, Paraná, Brazil; fernando.bmed@gmail.com; 4 Department of Dentistry, Ingá University Center, Uningá, Maringá 87035-510, Paraná, Brazil; dra.anacarbone@hotmail.com (A.C.C.); sventurajr.uff@gmail.com (S.V.); bryannenobree@gmail.com (B.B.d.S.N.); marialuizaboechat@hotmail.com (M.L.B.B.N.); mariana_mbcs@hotmail.com (M.B.C.-S.); 5 Department of Dentistry, State University of Maringa, Maringá 87035-510, Paraná, Brazil; rodrigopoluha@gmail.com; 6 Division of Oral Rehabilitation, Department of Dental Medicine, Karolinska Institutet, SE-14104 Huddinge, Sweden

**Keywords:** Poly-l-lactic acid, facial aesthetics, biostimulator, systematic review

## Abstract

The primary objective of this systematic review study was to investigate the effectiveness, durability, and adverse events of PLLA treatment for aesthetic indications. The search strategy was performed in MEDLINE (Ovid). The electronic literature search of five databases was performed, from the inception of the databases until the 12th of February 2024. This was to identify randomized clinical trials that assessed PLLA treatment in adult individuals exhibiting facial aging and/or facial lipoatrophy. Risk of bias was assessed using the Cochrane Risk-of-Bias Tool for Randomized Trials (RoB 2). Eleven RCTs out of 1467 identified citations were included. Four studies showed increased dermal thickness, significant improvement in facial lipoatrophy severity and aesthetic clinical scores, after PLLA treatment with its effects sustained for at least 25 months. Two studies demonstrated the superiority of PLLA over injectable human collagen. Also, three studies showed positive results favoring PLLA when compared with PH gel in lipoatrophy severity, transepidermal water loss, skin quality, elasticity, and patient satisfaction. All adverse events were mild-to-moderate in intensity, and the main ones worth noting were bruising, hematoma, tenderness, nodules, and edema. Five out of eleven studies were considered having high risk of bias. The evidence on the effectiveness and safety of PLLA for facial rejuvenation is of low quality; thus, the reported high effectiveness, safety, and long-lasting effects for this purpose should be further investigated.

## 1. Introduction

With the growing interest in facial rejuvenation and the consequent advancement in understanding the aging process, collagen bio-stimulators such us Poly-l-lactic Acid (PLLA) have been prominent in the aesthetic market [1]. PLLA is a synthetic, biodegradable, biocompatible, and immunologically inert polymer belonging to the family of alpha hydroxy acid polymers [2]. Its mechanism of action occurs through the stimulation of neo-collagenases by triggering a foreign body reaction to the injected material, resulting in a controlled cellular inflammatory response, which consequently activates fibroblasts to produce autologous collagen [3]. This results in a natural and semi-permanent correction of facial volume loss associated with aging [4].

Historically, PLLA was first approved in Europe as a filler in 1999, under the trade name New-Fill^®^ (Biotech Industry SA, Luxembourg). Then, in 2004, it was approved as Sculptra^®^ (Dermik Laboratories, Sanofi Aventis, Bridgewater, NJ, USA) by the FDA (Food and Drug Administration) as a lipoatrophy treatment for people with human immunodeficiency virus (HIV), presenting high efficacy in the restoration of facial volume loss [5,6,7]. In 2009, it was also approved for correction of deep nasolabial folds and other wrinkles in HIV population and recently, in 2023, an extension of the indications was approved for the correction of fine wrinkles in the cheek region. [8,9]. In addition, PLLA (Sculptra Aesthetic^®^), has also been used for aesthetic indications in healthy patients, showing an extensive track record of efficacy and safety [3,8,10].

Therefore, PLLA is a recommended treatment for enhancing skin tightness due to aging. It is also effective for improving wrinkles, creases, scars, and other changes caused by aging and volume loss. Application in various layers like the supraperiosteal, subcutaneous, and subdermal layers is advised for optimal results in facial rejuvenation by improving skin quality, firmness, and facial contour [11,12].

In addition, numerous clinical trials assessing its safety, effectiveness, and durability have been published. These studies included not only HIV populations, but also healthy individuals and reported that the majority of the studied volunteers were satisfied or very satisfied with the clinical results of PLLA treatment [6,8]. Notwithstanding these results, there is a necessity to assess the aforementioned studies, since most of them used different PLLA treatment protocols, mainly regarding the dilution process, the quantity of PLLA applied, and the treated region, factors that certainly influence the efficacy, durability, and adverse events of this bio-stimulator [13]. Additionally, it is of clinical importance to know the effectiveness and safety profile of PLLA compared with other collagen bio-stimulators available. Thus, there is a necessity of a systematic synthesis of PLLA literature for aesthetic indications, to enhance its uses in clinical practice and to provide a guideline protocol. Based on this, the aim of this systematic review was to investigate the effectiveness, durability, and adverse events of PLLA treatment for aesthetic indications.

## 2. Materials and Methods

### 2.1. Protocol and Research Question

This systematic review was registered beforehand in PROSPERO (the International Prospective Register of Systematic Reviews, #CRD42023472405). The research question was formed using the PICO framework [14], being an acronym for: P = Patients, I = Intervention, C = Comparison, O = Outcome. The population was comprised of patients (P) with signs of facial aging and/or lipoatrophy, while the investigated intervention (I) was treatment with Poly-l-Lactic Acid for the restoration of tissue volume and facial aging. The outcomes (O) were the efficacy, durability, and safety of PLLA, and the comparison (C) was among no treatment, placebo, or another treatment for the aging face and/or lipoatrophy. The present systematic review followed the Preferred Reporting Items for The PRISMA Extension Statement for Reporting of Systematic Reviews Incorporating Network Meta-Analyses of Health Care Interventions (the PRISMA-P checklist) [15] (Supplementary File S1).

The inclusion criteria were (a) randomized clinical trials; (b) adult individuals (over the age of 18) exhibiting facial aging; and (c) adult individuals (over the age of 18) presenting facial lipoatrophy. The following exclusion criteria were applied: (a) Studies that cannot be found in languages other than English, Portuguese, and Spanish; (b) publications irrelevant to the research question; and (c) cross-sectional, case-control, and observational studies, editorials, letters, legal cases, interviews, case-series, case reports, and reviews.

### 2.2. Search Strategy and Selection Criteria

The search strategy was created and executed in MEDLINE (Ovid) with the assistance of the librarians (LL) and (ELS) at the University Library at Karolinska Institutet. The search methods were reviewed by another librarian before the final searches. Together with authors RS and GDC, each search concept was identified using Medical Subject Headings (MeSH-terms) and free text terms. The Polyglot Search Translator [16] was utilized to translate the search into other databases. The final electronic search covered databases such as MEDLINE, EMBASE, CINAHL, and Web of Science from the inception of each database up to 12 February 2024. The duplication was carried out following the method by Bramer et al. (2016) [17]. Additionally, a step was included to compare digital object identifiers (DOI) and search the reference-lists of the included studies. The complete search strategy for all databases is provided in Supplementary File S2.

### 2.3. Selection of Studies

To mitigate any potential bias during the study screening process, we utilized the web-based tool Rayyan [18]. This screening was conducted independently and in a blinded fashion by authors RS and FCS. In instances of disagreement regarding eligibility, these were resolved through discussions with author GDC, who took the final decisions. Once all discrepancies were resolved, authors RS and FS endeavored to obtain the full texts of the included and potentially eligible studies. Subsequently, these retrieved studies were thoroughly reviewed in full text by authors RS and FCS to ascertain their alignment with the inclusion criteria. Any disagreements were addressed through discussions with author GDC, as before. Additionally, we identified supplementary articles through citation searches. The full texts of the identified studies were then retrieved and reviewed in the same manner as previously described.

### 2.4. Analysis of Risk of Bias

Risk of bias and quality assessment of the included articles were performed using the Cochrane Risk-of-Bias Tool for Randomized Trials (RoB 2) [19]. This tool is composed of five domains: randomization process (D1), deviations from the intended interventions (D2), missing outcome data (D3), measurement of the outcome (D4) and selection of the reported results (D5). Each domain can be judged for risk of bias into three categories: low risk, some concerns, or high risk. Two authors (AB and AC) evaluated the risk of bias for each study blinded and independently. In cases where conflict arose, it was resolved by discussion with the author RP, serving as a judge.

### 2.5. Extraction of Data

Following the risk of bias and quality assessment, data extraction was carried out. A data extraction form was devised, created (by authors RS, GDC), and pilot-tested independently on three randomly chosen studies by authors RS and MCS to ensure extraction consistency. The extracted data encompassed information on the characteristics of the included studies and participants, including author details, study type, diagnosis/criteria utilized, participant numbers, average age, gender distribution, doses of PLLA, dilution protocol, regions in which PLLA was injected, and details of randomized studies. Any discrepancies in the data extraction process were resolved by author GDC, acting as an adjudicator.

## 3. Results

### 3.1. Literature Search Outflow

**Figure 1:**
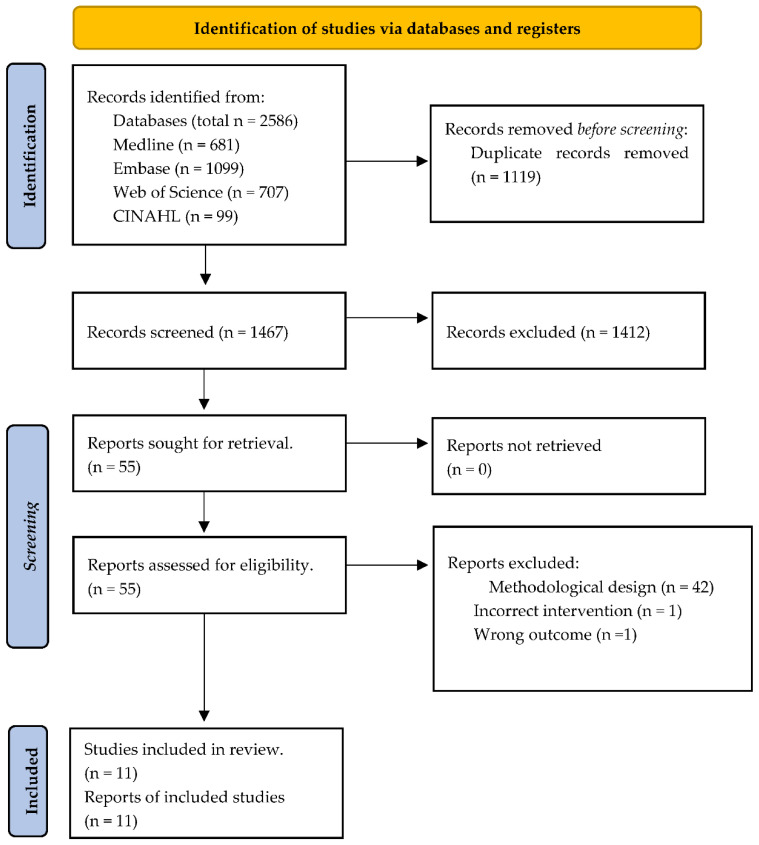
The figure illustrates the PRISMA flow chart of the database search strategy.

The entire literature search from all databases provided 2586 citations, of which 1119 were overlaps (Figure 1). Thus, 1467 citations were evaluated for eligibility. The titles and abstracts of those articles were then screened, which resulted in the exclusion of 1412 articles, leaving 55 full texts that were sought for retrieval, of which all of them were successfully retrieved. Out of these 55 full-texts, 44 full-texts did not meet the inclusion criteria and were excluded. Then, 11 full texts were included in this systematic review, all randomized controlled studies (RCTs) (Table 1).

**Table 1:** Table illustrating the extracted study characteristics of the eleven included studies.

Authors, Year	Population (P)	Intervention (I)	Comparison (C)	Outcomes (O)	Study Design (S)
Moyle, et al. 2004 [6]	N = 30 (15 in each group) Sex: 2 women; 28 men Age: mean 41.7 Subjects: HIV-related facial lipoatrophy	G1—immediate: three, PLLA (Newfill TM^®^) bilaterally injections session on day 1, 2 and 4 W later. Total, 4–5 mL/session D: not described F: 24 W A: facial photography, facial ultrasound, perceived changes in body shape (VAS) and HADS	G2—delayed: three, PLLA (Newfill TM^®^) bilaterally injections sessions on W 12, 14 and 16. Total, 4–5 mL. per session	A significant increase in dermal thickness in injected regions and in self-assessment scores was found in G1 compared to G2 at week 12. No differences in dermal thickness were observed between the groups at week 24. The severity score of lipoatrophy declined in both groups. AE: bruising and limited superficial local cellulitis.	Randomized, open label
Moyle, et al. 2006 [20]	N = 27 (G1: n = 13; G2: n = 14) Sex: 2 women; 25 men mean Age: mean 41 years Subjects: HIV-related facial lipoatrophy	G1—immediate: PLLA (Newfill TM^®^), three bilaterally injections session on 1, 2 and 4 W. Total, 4–5 mL/session D: 2 mL/sterile water and 1 mL/lidocaine F: 2 years A: perceived changes in body shape (VAS) and HADS	G2—delayed: three, PLLA (Newfill TM^®^) bilaterally injections sessions on 12, 14 and 16 W. Total, 4–5 mL/session	Patients’ self-perceived facial thinness was significantly more positive after 2 years than at baseline in both groups. Also, both groups were less depressed and anxious at the 2 years recall visit than at baseline. However, these improvements only reached statistical significance for depression in the delayed group. AE: From baseline to 2 years, 112 treatment AEs were reported and classified as mild, moderate and severe in intensity in 44, 47 and 7% of cases, respectively. A single case of injection-site induration and nine cases of injection-site nodules were noted at the 2 years recall.	Randomized, open label
Carey, et al. 2007 [5]	N = 100 (G1 n = 50; G2 n = 50) Sex: 8 women; 92 men Age: mean 49.8 Subjects: HIV-related facial lipoatrophy and immunocompetent	G1—immediate: PLLA 4 bilaterally injection sessions at 2 W interval. 5 mL/side/session D: 5 mL/sterile water F: −1, 1, 3, 5, 7, 12 and 24 W A: CT and MBSRQ-AS	G2—delayed: PLLA bilaterally injections after 24 W	PLLA did not increase FSTV. At 24 W FSTV scores do not differ significantly between groups. Facial lipoatrophy severity was improved in (90%) in G1 compared with 18% G2 at 12 and 24 W. MBSRQ-AS scores were improved in G1 compared with G2 at 12 and 24 W. AE: 96% of patients experienced at least 1 procedure/product-related AE, like pain/discomfort, localized edema, and erythema. Most events were grade mild or moderate and of short duration. At 24 W, noninflammatory nodules and papule were reported in 6 participants.	Randomized, open label, multicenter
Brandt, et al. 2010 [2]	N = 233 Sex: 220 women; 13 men Age: mean 51.4 Subjects: Immunocompetent	G1: PLLA (Sculptra^®^), 1 to 4 bilaterally injection sessions at 3 W interval. Maximum of 2.5 mL/side or 5 mL total/session D: 5 mL/sterile water, 2 h before injection F: 3 W; 3, 6, 9, 13 M A: IGE	G2: Human collagen (CosmoPlast^®^), 1 to 4 bilaterally injection sessions of 1 mL/side, at 3 W intervals	G1 reported a significantly higher improvement in IGE through all the follow-ups, compared to G2. AE: G1 demonstrated a favorable safety and tolerability profile comparable to G2. The majority of AE were of mild to moderate in intensity (papules and nodules).	Randomized, single-blinded, multicenter
Narins, et al. 2010 [10]	N = 233 (G1: n = 116; G2: n = 117) Sex: 220 women; 13 men Age: mean 51.4 years Subjects: Immunocompetent	G1: PLLA (Sculptra^®^), 1 to 4 bilaterally injection sessions at 3 W interval. Maximum of 2.5 mL/side or 5 mL total/session D: 5 mL/sterile water, 2 h before injection F: 3 W; 3, 6, 9, 13,19 and 25 M A: Photography and WAS scale	G2: Human collagen (CosmoPlast^®^), 1 to 4 bilaterally injection sessions of 1 mL/side at 3 W intervals	A significant improvement in the mean change from baseline in WAS score was reported for G1 compared to G2 at 13-month follow-up. Improvements (up to 25 months) were significantly greater just in G1. AE: The majority were mild to moderate in intensity and self-limiting. Higher incidence of AEs was reported on the collagen group (63.2%) compared with the injectable PLLA group (53.4%).	Randomized, single-blinded, multicenter
Brown, et al. 2011 [21]	N = 233 (G1 n = 116; G2 n = 117) Sex: 220 Women; 13 men Age: mean 51.4 Subjects: nasolabial fold wrinkles	G1: PLLA (Sculptra^®^), 1 to 4 bilaterally injection sessions at 3 W interval each. Maximum of 2.5 mL/side or 5 mL total/session D: 5 mL/sterile water, 2 h before injection F: 3 W; 3, 6, 9, 13,19 and 25 M A: SGE, SS	G2: Human collagen (CosmoPlast^®^), 1 to 4 bilaterally injection sessions of 1 mL/side at 3 W intervals each	Aesthetic improvement in G1 was maintained overall above 90% at the 13 M follow-up and above 81% at 19 and 25 M follow-ups. In contrast, G2 declined to 15% at 13 M. In G1, the proportion of subjects with good to excellent SS scores remained above 84% throughout the follow-up periods, while in G2, subjects with good to excellent SS scores decreased progressively until the 13 M follow-up. AE: The most common AEs were injection-site erythema, pain and pruritus in both groups. Product-related application-site papules and nodules were found in both groups after 13 M, decreasing in G1 at 25 M. All events were of mild to moderate intensity.	Randomized, parallel, multicenter
Lafaurie, et al. 2012 [22]	N = 148 (G1: n = 73; G2: n = 75) Sex: 10 women; 138 men Age: mean 47 years Subjects: HIV-related facial lipoatrophy	G1: PLLA (Newfill TM^®^), 3 to 7 injection sessions of every 4 W until 24 W D: 4 mL/sterile water and 1 mL/lidocaine, 2 h before injections F: 96 W A: VAS, photographic images, MOS-HIV	G2: PH (Eutrophill^®^) gel	A significant improvement from baseline in lipoatrophy severity in both groups at week 48 and 96 was found. No significant differences were found in patients’ satisfaction scores and lipoatrophy severity between treatments at 48 and 96 weeks. AE: Bleeding, hematoma at injection site, vagal hypertonia during injections, and edema post-injections were reported in both groups and considered mild or moderate. Subcutaneous nodules were reported in 41% and 37% of cases in the PLLA and PH.	Prospective, randomized, single-blinded non inferiority
Bohnert, et al. 2019 [3]	N = 40 (G1 n = 20; G2 n = 20) Sex: women Age: 30–60 Subjects: shallow to deep nasolabial fold, other facial wrinkles	G1: PLLA (Sculptra Aesthetic^®^) 3 bilaterally injection session at intervals of 4 W. 5 to 6 mL/side/session. D: 5 mL/sterile water 24 h before injections, after this, 2 mL/sterile water. Immediately before injections, 2 mL/1% lidocaine was added. Total of 9 mL. F: 6, 9 and 12 M A: Skin Quality Rating (VAS), Subject Satisfaction, Standardized Photography, Skin Physiology (Corneometer, Tewameter and Cutometer).	G2: Saline Solution	All skin quality assessments were significantly improved at the 12 M visit in G1 compared with G2. Patient satisfaction was higher in G1 compared with G2 through all the study. At the 12 M follow-up, the G1 exhibited a greater reduction in trans epidermal water loss compared to G2. Elasticity significantly increased in G1 compared with G2 in all follow-ups. AE: No adverse events related to the treatment were reported. Temporary mild swelling was reported in three patients.	Randomized, controlled, double-blinded, multicenter
Palm, et al. 2021 [23]	N = 80 Sex: 76 women; 4 men Aged: mean 51.5 Subjects: deficiencies in nasolabial contour	G1: PLLA (Sculptra Aesthetic^®^), up to 4 injections sessions with 4 W intervals. Maximum volume 9 mL/session (4.5 mL per nasolabial fold). D: 8 mL/sterile water and 1 mL of 2% lidocaine hydrochloride. Total volume of 9 mL. No standing time was required. F: 16, 24, 32, 40 and 48 W A: WAS, GAIS, FACE-Q and a subject questionnaire.	G2: PLLA (Sculptra Aesthetic^®^), up to 4 injections sessions with 4 W intervals. Maximum volume 5 mL/session (2.5 mL per nasolabial fold). D: 5 mL/sterile water. Two to 72 h standing time.	WAS scores were slightly higher for G2 than for G1 at earlier time points; however, values were similar between the groups at 48 W. GAIS scores showed that all subjects improved at all visits. Individuals in both groups were satisfied with the appearance of their nasolabial folds after 48 W. AE: 11.9% in G1 and 33.3% in G2 reported AEs related to the study product or injection procedure. The most common AEs were headache, rhinorrhea, and perioral hypoesthesia, all of mild intensity in the treatment group.	Randomized, single-blinded, multicenter
Han, et al. 2023 [24]	N = 55 (G1 n = 55; G2 n = 55) Sex: 48 women; 7 men Age: mean 53.8 Subjects: nasolabial folds wrinkles	G1: PLLA (Sculptra) a maximum of 1 mL. Optionally, touch-up injection at 6 W D: 8 mL/sterile water 12 h before injection. F: 0, 2 W, 3 and 6 M A: WSRS score, and GAIS	G2: PLLA (GANA V), a maximum of 1 mL. Optionally, touch-up injection at 6 W D:15 mL/sterile	WSRS scores showed a significant improvement for G2 and no improvements in G1 after 6 months. No significant differences were observed between the two groups in GAIS scores in all follow-ups. A higher satisfaction score in the immediate outcome assessment was reported in G2 compared with G1. G2 has an acceptable 6 M effectiveness compared with G1, which is in line with the established non-inferiority margin AE: Both groups experienced erythema, tenderness, firmness, swelling, bumps, bruising, and pigmentation. There were no significant differences in the rate of injection site reactions between the two groups.	Randomized, double-blind, non-inferiority, split face controlled
Fabi, et al. 2024 [8]	N = 149 (G1 n = 97; G2 n = 52) Sex: (144 women, 5 men) Age: mean 60.7 Subjects: moderate to severe cheek wrinkles.	G1: PLLA-SCA (Sculptra^®^) 1 bilaterally injection session, with 3 more injection sessions permitted at monthly intervals. D: 8 mL/ sterile water and 1 mL/lidocaine (2%) F: 7, 9, and 12 M A: GCWS, GAIS, and FACE-Q questionnaire	G2: No treatment	GCWS scores at rest and dynamic were significantly higher in G1 compared to G2 at 7, 9, 12 M GAIS scores showed responders to treatment just in G1 through all the study. FACE-Q scores showed a significant increase in G1 in all follow-up points when compared with G2. AE: The most common post-treatment symptoms were tenderness, bruising, swelling, and pain, being mild to moderate in intensity. The most common treatment-related AEs included injection site bruising, dizziness, and headache. No serious treatment-related AEs were reported.	Randomized, single-blinded, no-controlled

D: dilution; F: follow-up; A: assessments; AE: Adverse events; PLLA: Poly-l-Lactic Acid Filler; PH: Polyacrylamide hydrogel; MOS-HIV: Medical outcomes study HIV health survey; HADS: Hospital Anxiety and Depression Scale (HADS); VAS: Visual Analogue Scale; WAS: Wrinkle Assessment Scale; IGE: Investigator Global Evaluation; MBSRQ-AS: Multidimensional Body–Self Relations Questionnaire-Appearance Scales; FSTV: Facial Soft Tissue Volume; GAIS: Global Aesthetic Improvement Scale; GCWS: Galderma Cheek Wrinkles Scale; SGE: Subject Global Evaluation; SS: Subject Satisfaction; WSRS: Wrinkle Severity Rating Scale; CT: computerized tomography; W: weeks; M: months.

### 3.2. Studies’ Results

#### 3.2.1. Efficacy and Durability of PLLA: Immediate × Delayed Protocol

Some studies used an immediate or delayed protocol for PLLA injection. As for short follow-up (up to 12 weeks), some studies found a significant increase in dermal thickness in injected regions and in self-assessment scores for the immediate group, compared to the delayed group [6], but no differences at week 24. The severity score of lipoatrophy declined in both groups after 2 years [6], and patients’ self-perceived facial thinness was significantly more positive for both groups, leading to less depression and anxiety; moreover, PLLA’s positive psychological and physical effects persisted for at least 18 months [20]. Conversely, some studies found a significant improvement in facial lipoatrophy severity [5], MBSRQ-AS scores [5], aesthetic improvement [21], and SS scores [21] for the participants in the immediate group protocol. In addition, Brown et al. [21] found that individuals receiving injectable PLLA maintained aesthetic improvement for up to 25 months.

#### 3.2.2. PLLA × Human Collagen

Two studies compared the use of PLLA with injectable human collagen. In both, the PLLA demonstrated statistically significant improvements from baseline in WAS score^2^ and in IGE through all the follow-ups [10], being superior to human collagen.

#### 3.2.3. Other Comparisons

PLLA was compared with polyacrylamide hydrogel (PH) gel by Lafaurie et al. (2012) [22]. A significant improvement from baseline in lipoatrophy severity was found in both groups at week 48 and 96, with no significant differences in patients satisfaction scores [22]. When compared to saline solution (placebo), participants receiving PLLA had significantly improved skin quality at the 12-month visit and greater reduction in transepidermal water loss, higher elasticity, and higher satisfaction through all the follow-ups [3]. Moreover, this improvement was also found when comparing PLLA with no treatment [8]. Participants receiving PLLA had higher GCWS scores at rest and dynamic, increased GAIS scores, and significantly increased FACE-Q scores in all follow-ups [8].

#### 3.2.4. Adverse Events

Most studies reported mild-to-moderate intensity adverse events, such as bruising, bleeding, tenderness, firmness, injection-site hematoma, erythema, pain, pigmentation and pruritus, limited superficial local cellulitis, headache, rhinorrhea, perioral hypoesthesia, dizziness, vagal hypertonia during injections, and edema post-injections [5,6,8,21,22,23,24]. Additionally, Moyle et al. [20] reported that 7% of adverse events were severe in intensity: one case of injection-site induration and nine cases of injection-site nodules, which were noted at the 2-year recall. Subcutaneous papules and nodules were also reported in shorter follow-ups in some studies [5,10,21], being presented in 41% of the studied population in the study conducted by Lafaurie et al. [22].

Studies comparing PLLA and human collagen concluded that a higher incidence of adverse events was reported on the collagen groups compared with the injectable PLLA groups [2,10]. Further, only one study reported no adverse events related to the treatment [3], stating that just three patients had temporary mild swelling.

#### 3.2.5. Dilution Protocol

Only one study tested the dilution protocol for PLLA (Sculptra). Palm et al. [23] evaluated two protocols to dilute PLLA: 8 mL/sterile water + 1 mL of 2% lidocaine hydrochloride, totaling 9 mL of product, with no standing time required (experimental—G1). The control group (G2) received PLLA diluted in 5 mL of sterile water only, with a standing time from 2 to 72 h. The amount of product injected was also different: for G1, 4.5 mL per nasolabial fold, and as for G2, 2.5 mL per nasolabial fold. In the last follow-up (48 weeks), both groups presented similar results for WAS, GAIS, and satisfaction with results. The remaining studies presented varied protocols according to what was prescribed by manufacturers by the time of data collection (which has changed over the years).

#### 3.2.6. Quality Assessment

None of the included studies were considered as having low risk of bias. Five articles were considered as having high risk of bias by not fulfilling the criteria for the topic “deviation from intended intervention” (D2) [5,6,8,20,23]. The risk of bias of the remaining six articles was considered as some concerns [2,3,10,21,22,24]. Further, most of the studies presented some concerns regarding the “randomization process” (D1) and for “selection of the reported of results” (D5) all manuscripts were considered as presenting “some concerns” related to the risk of bias (Table 2).

## 4. Discussion

The primary findings of this systematic review indicate that PLLA is a highly effective and long-lasting treatment for facial aesthetics. Its effectiveness surpasses all other substances it was compared to, being a safe treatment, as most adverse effects were mild to moderate and resolved spontaneously. However, it is important to note that these results are mainly based on low-quality evidence.

The reported high efficacy of PLLA in all the included studies could be explained by its composition and mechanism of action that favors neocollagenesis. PLLA is an enantiomeric polymer of lactic acid, that is part of the alpha-hydroxy acids family, being biocompatible and biodegradable in nature. Its particles have an average size of 52 µm (40 µm to 63 µm) and are plate-shaped and non-porous, which accounts for their lower degradation rate when injected into tissues [25,26]. This slower degradation favors the process of neocollagenesis for a longer period. This process begins with the immune cells recognizing PLLA particles as foreign bodies resulting in a controlled cellular inflammatory response, in which monocytes are recruited and transform into macrophages, then fuse to form giant cells, recruit fibroblasts, and increase the levels of TGF-β1 and tissue inhibitor of metalloproteinase 1 (TIMP1), promoting the deposition of type I and III collagen. This process contributes to increase collagen production in the area treated with PLLA, resulting in firmer, thicker, and more elastic skin [25,26].

PLLA is made available in the form of a lyophilized powder in a sterile vial also containing non-pyrogenic mannitol (which improves the lyophilization of the particles), croscarmellose (an emulsifying agent that maintains particle distribution after reconstitution) [26]. Over the years, the process of reconstituting PLLA particles has been revisited [27]. In addition, most of the studies included in this review presented varied reconstitution protocols. However, it is important to highlight that the reconstitution instructions of PLLA have changed according to the time when the studies were performed, following the manufacturer’s instructions at each specific time. Initially, with NewFill^®^/Sculptra^®^, reconstitutions were carried out with a total volume of 5 mL [27]. In the present study, the most common reconstitution protocols were using 5 mL or 8 mL of sterile water. Notwithstanding these differences, when these reconstitution protocols were compared no significant differences were found in the efficacy of PLLA [23]. Additionally, the efficacy remains unchanged whether reconstituted immediately or 24 to 72 h before the procedure [27]. The resting period of 24 to 72 h was considered necessary to hydrate the PLLA molecules, forming a homogeneous suspension with carboxymethylcellulose (CMC) without the formation of lumps. However, recent studies have shown no statistical differences between performing the reconstitution at 72 h and immediately, without altering clinical efficacy. Therefore, based on the results of the included studies, we might confirm that the efficacy of PLLA is not altered by both the reconstituted volume and reconstitution time before injection procedure [27].

Since PLLA particles are not amorphous, they easily agglomerate with the excipient CMC, commonly leading to needle and cannula blockages making the injection process difficult and the formation of nodules after application, which was one of the most common adverse effects related to the product found in this study. To prevent nodules forming, post-application massages were recommended to avoid these complications, as the mechanical process dissolves potential nodules that may form [3]. The chance of nodule dissolution is higher when performed after application, even though a proper protocol has not been reported. Importantly, the most prevalent adverse effects reported in this review are related to the injection procedure and patients’ following post-injection recommendations, and not to PLLA per se. Furthermore, despite the fibroplasia process caused by PLLA influencing aesthetic outcomes, there is no evidence of residual fibrosis [4], insignificant amounts of degradation residues are found in vital organs, and the product is completely eliminated within 18 months, which demonstrates its safety [28]. However, only one study was considered free of treatment-related adverse events [3].

The global market for PLLA is undergoing substantial growth. In 2023, the market size for PLLA fillers was estimated to be approximately $268.1 million, with Scultra^®^ being the front leader in the market [29]. In addition, among the included studies, all were conducted with the commercial PLLA product Sculptra^®^/NewFill^®^, confirming the predominance of these products in the available data. Only one study used a different product, GANA V^®^, to verify non-inferiority, compared with Sculptra^®^ [24]. Although both products are composed of PLLA, they have different physicochemical properties, resulting in distinct particle shapes which can influence their clinical responses, durability, and adverse effects. Due to the limited literature on GANA V^®^, more studies are needed to prove its efficacy, durability, and safety, as well as to evaluate its polymeric chemical composition.

The quality of the evidence presented in this systematic review should be carefully considered when interpreting the results. Although PLLA has demonstrated significant efficacy and an acceptable safety profile compared to other substances, most of the included studies were classified as having high or moderate concerns regarding the risk of bias. Additionally, the lack of consistency in evaluation methods and clinical protocols limit the robustness of the conclusions. Therefore, even though PLLA treatment for facial aesthetic is increasing in clinical practice, it is essential to develop higher-quality clinical trials including objective and subjective assessments to confirm the findings presented in this systematic review.

### 4.1. Study Strengths and Limitations

The following measures were taken in order to minimize bias. The literature search was performed in several databases with the help of search experts. The article selection process and the risk of bias assessment were performed by two blinded authors independently to prevent bias, ensuring that each other’s decisions would not be a factor of influence. Another strength of this systematic review is the inclusion of only RCTs. However, this could also be seen as a limitation, as the included RCTs exhibit methodological flaws that prevent them from being considered high quality. Also, a quantitative analysis was not feasible since the outcomes and assessment of the included studies were diverse. In addition, there were substantial variations in PLLA reconstitution and administration protocols, which impacted the comparability of results. Also, the relatively low number of included studies (11) could be considered a limitation for this review.

### 4.2. Clinical Implications and Generalizability

The results of this study indicate that the clinical response to PLLA in both HIV patients and general patients for aesthetic purposes seems to be high. Both professional and patient evaluations are positive in almost all studies, indicating a significant improvement in dermal thickness and aesthetic perception. However, it is necessary to interpret these results with caution since all studies demonstrate gaps that reveal strong risks of bias, preventing a rigorous assessment of the real effects of PLLA-based biopolymers and complete recommendations for clinical practice. Additionally, it might be possible that conditions that were not studied in the included studies, like autoimmune and connective tissue diseases and the presence of permanent fillers, influence the aesthetic results of PLLA treatment.

### 4.3. Future Applications

To confirm the efficacy and safety of PLLA in facial aesthetic treatments, future high-quality studies should be conducted with high methodological rigor and impartiality. These studies should adopt standardized methodologies and ensure proper randomization and blinding processes, which were the major limitations of the included studies. These recommendations are necessary to reduce the risks of bias in future RCTs and to establish validated clinical protocols to provide a robust and reliable evidence base on the clinical effects of PLLA.

## 5. Conclusions

PLLA has been demonstrated to be a highly effective and long-lasting treatment for facial aesthetics with a reasonable safety profile. However, all findings are supported by low-quality evidence.

## Figures and Tables

**Table 2:** Table summarizing quality assessment and risk of bias.

Author, Year	Randomization Process	Deviations from Intended Interventions	Missing Outcome Data	Measurement of the Outcome	Selection of the Reported Results	Overall
Moyle et al., 2004 [6]	Some concerns	High risk	Low risk	Low risk	Some concerns	High risk
Moyle et al., 2006 [20]	Some concerns	High risk	Some concerns	Some concerns	Some concerns	High risk
Carey et al., 2007 [5]	Some concerns	High risk	Low risk	Low risk	Some concerns	High risk
Brandt et al., 2010 [2]	Some concerns	Low risk	Some concerns	Some concerns	Some concerns	Some concerns
Narins et al., 2011 [10]	Some concerns	Some concerns	Low risk	Low risk	Some concerns	Some concerns
Brown et al., 2011 [21]	Low risk	Some concerns	Some concerns	Some concerns	Some concerns	Some concerns
Lafaurie et al., 2013 [22]	Low risk	Some concerns	Some concerns	Some concerns	Some concerns	Some concerns
Bohnert et al., 2019 [3]	Low risk	Low risk	Some concerns	Low risk	Some concerns	Some concerns
Palm et al., 2021 [23]	Some concerns	High risk	Low risk	Low risk	Some concerns	High risk
Han et al., 2023 [24]	Low risk	Low risk	Some concerns	Low risk	Some concerns	Some concerns
Fabi et al., 2024 [8]	Some concerns	High risk	Low risk	Low risk	Some concerns	High risk

## Data Availability

The datasets generated during and/or analyzed during the current study are not publicly available but are available from the corresponding author on reasonable request.

## Supplementary Materials

The following supporting information can be downloaded at: https://www.mdpi.com/article/10.3390/polym16182564/s1, S1: Prisma Checklist; S2: Search strategy in different databases. Reference [30] is cited in the supplementary materials.

## References

[1] Christen M.-O. (2022). Collagen stimulators in body applications: A review focused on poly-l-lactic acid (PLLA). Clin. Cosmet. Investig. Dermatol..

[2] Narins R.S., Baumann L., Brandt F.S., Fagien S., Glazer S., Lowe N.J., Monheit G.D., Rendon M.I., Rohrich R.J., Werschler W.P. (2010). A randomized study of the efficacy and safety of injectable poly-l-lactic acid versus human-based collagen implant in the treatment of nasolabial fold wrinkles. J. Am. Acad. Dermatol..

[3] Bohnert K., Dorizas A., Lorenc P., Sadick N.S. (2019). Randomized, controlled, multicentered, double-blind investigation of injectable poly-l-lactic acid for improving skin quality. Dermatol. Surg..

[4] Fitzgerald R., Vleggaar D. (2011). Facial volume restoration of the aging face with poly-l-lactic acid. Dermatol. Ther..

[5] Carey D.L., Baker D., Rogers G.D., Petoumenos K., Chuah J., Easey N., Machon K., Cooper D.A., Emery S., Carr A. (2007). A randomized, multicenter, open-label study of poly-l-lactic acid for HIV-1 facial lipoatrophy. JAIDS J. Acquir. Immune Defic. Syndr..

[6] Moyle G., Lysakova L., Brown S., Sibtain N., Healy J., Priest C., Mandalia S., Barton S. (2004). A randomized open-label study of immediate versus delayed polylactic acid injections for the cosmetic management of facial lipoatrophy in persons with HIV infection. HIV Med..

[7] Lam S.M., Azizzadeh B., Graivier M. (2006). Injectable poly-l-lactic acid (Sculptra): Technical considerations in soft-tissue contouring. Plast. Reconstr. Surg..

[8] Fabi S., Hamilton T., LaTowsky B., Kazin R., Marcus K., Mayoral F., Joseph J., Hooper D., Shridharani S., Hicks J. (2024). Effectiveness and Safety of Sculptra Poly-l-Lactic Acid Injectable Implant in the Correction of Cheek Wrinkles. J. Drugs Dermatol. JDD.

[9] Food and Drug Administration FDA (2023). Summary of Safety and Effectiveness Data (SSES). https://www.accessdata.fda.gov/cdrh_docs/pdf16/P160035B.pdf.

[10] Brandt F.S., Cazzaniga A., Baumann L., Fagien S., Glazer S., Kenkel J.M., Lowe N.J., Monheit G.D., Narins R.S., Rendon M.I. (2011). Investigator global evaluations of efficacy of injectable poly-l-lactic acid versus human collagen in the correction of nasolabial fold wrinkles. Aesthetic Surg. J..

[11] Haddad A., Kadunc B.V., Guarnieri C., Noviello J.S., da Cunha M.G., Parada M.B. (2017). Conceitos atuais no uso do ácido poli-l-láctico para rejuvenescimento facial: Revisão e aspectos práticos. Surg. Cosmet. Dermatol..

[12] Akinbiyi T., Othman S., Familusi O., Calvert C., Card E.B., Percec I. (2020). Better results in facial rejuvenation with fillers. Plast. Reconstr. Surg. Glob. Open.

[13] Butterwick K., Lowe N.J. (2009). Injectable poly-l-lactic acid for cosmetic enhancement: Learning from the European experience. J. Am. Acad. Dermatol..

[14] Riva J.J., Malik K.M., Burnie S.J., Endicott A.R., Busse J.W. (2012). What is your research question? An introduction to the PICOT format for clinicians. J. Can. Chiropr. Assoc..

[15] Hutton B., Salanti G., Caldwell D.M., Chaimani A., Schmid C.H., Cameron C., Ioannidis J.P., Straus S., Thorlund K., Jansen J.P. (2015). The PRISMA extension statement for reporting of systematic reviews incorporating network meta-analyses of health care interventions: Checklist and explanations. Ann. Intern. Med..

[16] Clark J.M., Sanders S., Carter M., Honeyman D., Cleo G., Auld Y., Booth D., Condron P., Dalais C., Bateup S. (2020). Improving the translation of search strategies using the Polyglot Search Translator: A randomized controlled trial. J. Med. Libr. Assoc. JMLA.

[17] Bramer W.M., Giustini D., de Jonge G.B., Holland L., Bekhuis T. (2016). De-duplication of database search results for systematic reviews in EndNote. J. Med. Libr. Assoc. JMLA.

[18] Ouzzani M., Hammady H., Fedorowicz Z., Elmagarmid A. (2016). Rayyan—A web and mobile app for systematic reviews. Syst. Rev..

[19] Chandler J., Cumpston M., Li T., Page M.J., Welch V. (2019). Cochrane Handbook for Systematic Reviews of Interventions.

[20] Moyle G.J., Brown S., Lysakova L., Barton S.E. (2006). Long-term safety and efficacy of poly-l-lactic acid in the treatment of HIV-related facial lipoatrophy. HIV Med..

[21] Brown S.A., Rohrich R.J., Baumann L., Brandt F.S., Fagien S., Glazer S., Kenkel J.M., Lowe N.J., Monheit G.D., Narins R.S. (2011). Subject global evaluation and subject satisfaction using injectable poly-l-lactic acid versus human collagen for the correction of nasolabial fold wrinkles. Plast. Reconstr. Surg..

[22] Lafaurie M., Dolivo M., Girard P.M., May T., Bouchaud O., Carbonnel E., Madelaine I., Loze B., Porcher R., Molina J.M. (2013). Polylactic acid vs. polyacrylamide hydrogel for treatment of facial lipoatrophy: A randomized controlled trial [Agence Nationale de Recherches sur le SIDA et les Hépatites Virales (ANRS) 132 SMILE]. HIV Med..

[23] Palm M., Weinkle S., Cho Y., LaTowsky B., Prather H. (2021). A Randomized Study on PLLA Using Higher Dilution Volume and Immediate Use Following Reconstitution. J. Drugs Dermatol..

[24] Han W.Y., Kim H.J., Kwon R., Kang S.M., Yon D.K. (2023). Safety and efficacy of Poly-l-Lactic acid filler (Gana V vs. Sculptra) injection for correction of the nasolabial fold: A double-blind, non-inferiority, randomized, split-face controlled trial. Aesthetic Plast. Surg..

[25] Oh S., Lee J.H., Kim H.M., Batsukh S., Sung M.J., Lim T.H., Lee M.H., Son K.H., Byun K. (2023). Poly-l-lactic acid fillers improved dermal collagen synthesis by modulating m2 macrophage polarization in aged animal skin. Cells.

[26] Sedush N.G., Kalinin K.T., Azarkevich P.N., Gorskaya A.A. (2023). Physicochemical characteristics and hydrolytic degradation of polylactic acid dermal fillers: A comparative study. Cosmetics.

[27] Baumann K., Alm J., Norberg M., Ejehorn M. (2020). Immediate Use after Reconstitution of a Biostimulatory Poly-l-Lactic Acid Injectable Implant. J. Drugs Dermatol. JDD.

[28] Gupta A., Kumar V. (2007). New emerging trends in synthetic biodegradable polymers–Polylactide: A critique. Eur. Polym. J..

[29] Riddhesh D. (2023). Global Poly-l-Lactic Acid (PLLA) Filler Market. https://dataintelo.com/report/global-poly-l-lactic-acid-plla-filler-market/.

[30] Page M.J., McKenzie J.E., Bossuyt P.M., Boutron I., Hoffmann T.C., Mulrow C.D., Shamseer L., Tetzlaff J.M., Akl E.A., Brennan S.E. (2021). The PRISMA 2020 statement: An updated guideline for reporting systematic reviews. BMJ.

