# Supplementary material for: Efficacy and Safety of Poly-l-Lactic Acid in Facial Aesthetics: A Systematic Review
Source: Polymers (Basel). 2024 Sep 11;16(18):2564. doi: 10.3390/polym16182564 (PMC11435306; doi:10.3390/polym16182564)
Supplement: Supplementary file 1 [file polymers-16-02564-s001.zip › polymers-3134447-supplementary.pdf]

Section and Topic	Item #	Checklist item	Location where item is reported
**TITLE**			
Title	1	Identify the report as a systematic review.	1
**ABSTRACT**			
Abstract	2	See the PRISMA 2020 for Abstracts checklist.	2
**INTRODUCTION**			
Rationale	3	Describe the rationale for the review in the context of existing knowledge.	3-4
Objectives	4	Provide an explicit statement of the objective(s) or question(s) the review addresses.	4
**METHODS**			
Eligibility criteria	5	Specify the inclusion and exclusion criteria for the review and how studies were grouped for the syntheses.	4-5
Information sources	6	Specify all databases, registers, websites, organisations, reference lists and other sources searched or consulted to identify studies. Specify the date when each source was last searched or consulted.	5
Search strategy	7	Present the full search strategies for all databases, registers and websites, including any filters and limits used.	5
Selection process	8	Specify the methods used to decide whether a study met the inclusion criteria of the review, including how many reviewers screened each record and each report retrieved, whether they worked independently, and if applicable, details of automation tools used in the process.	6
Data collection process	9	Specify the methods used to collect data from reports, including how many reviewers collected data from each report, whether they worked independently, any processes for obtaining or confirming data from study investigators, and if applicable, details of automation tools used in the process.	6
Data items	10a	List and define all outcomes for which data were sought. Specify whether all results that were compatible with each outcome domain in each study were sought (e.g. for all measures, time points, analyses), and if not, the methods used to decide which results to collect.	7
	10b	List and define all other variables for which data were sought (e.g. participant and intervention characteristics, funding sources). Describe any assumptions made about any missing or unclear information.	7
Study risk of bias assessment	11	Specify the methods used to assess risk of bias in the included studies, including details of the tool(s) used, how many reviewers assessed each study and whether they worked independently, and if applicable, details of automation tools used in the process.	6
Effect measures	12	Specify for each outcome the effect measure(s) (e.g. risk ratio, mean difference) used in the synthesis or presentation of results.	--
Synthesis methods	13a	Describe the processes used to decide which studies were eligible for each synthesis (e.g. tabulating the study intervention characteristics and comparing against the planned groups for each synthesis (item #5)).	--
	13b	Describe any methods required to prepare the data for presentation or synthesis, such as handling of missing summary statistics, or data conversions.	--

Section and Topic	Item #	Checklist item	Location where item is reported
	13c	Describe any methods used to tabulate or visually display results of individual studies and syntheses.	--
	13d	Describe any methods used to synthesize results and provide a rationale for the choice(s). If meta-analysis was performed, describe the model(s), method(s) to identify the presence and extent of statistical heterogeneity, and software package(s) used.	--
	13e	Describe any methods used to explore possible causes of heterogeneity among study results (e.g. subgroup analysis, meta-regression).	--
	13f	Describe any sensitivity analyses conducted to assess robustness of the synthesized results.	--
Reporting bias assessment	14	Describe any methods used to assess risk of bias due to missing results in a synthesis (arising from reporting biases).	6
Certainty assessment	15	Describe any methods used to assess certainty (or confidence) in the body of evidence for an outcome.	--
**RESULTS**			
Study selection	16a	Describe the results of the search and selection process, from the number of records identified in the search to the number of studies included in the review, ideally using a flow diagram.	7
	16b	Cite studies that might appear to meet the inclusion criteria, but which were excluded, and explain why they were excluded.	7
Study characteristics	17	Cite each included study and present its characteristics.	7-10
Risk of bias in studies	18	Present assessments of risk of bias for each included study.	10
Results of individual studies	19	For all outcomes, present, for each study: (a) summary statistics for each group (where appropriate) and (b) an effect estimate and its precision (e.g. confidence/credible interval), ideally using structured tables or plots.	7-8
Results of syntheses	20a	For each synthesis, briefly summarise the characteristics and risk of bias among contributing studies.	9
	20b	Present results of all statistical syntheses conducted. If meta-analysis was done, present for each the summary estimate and its precision (e.g. confidence/credible interval) and measures of statistical heterogeneity. If comparing groups, describe the direction of the effect.	--
	20c	Present results of all investigations of possible causes of heterogeneity among study results.	--
	20d	Present results of all sensitivity analyses conducted to assess the robustness of the synthesized results.	--
Reporting biases	21	Present assessments of risk of bias due to missing results (arising from reporting biases) for each synthesis assessed.	--
Certainty of	22	Present assessments of certainty (or confidence) in the body of evidence for each outcome assessed.	--

Section and Topic	Item #	Checklist item	Location where item is reported
evidence			
**DISCUSSION**			
Discussion	23a	Provide a general interpretation of the results in the context of other evidence.	10
	23b	Discuss any limitations of the evidence included in the review.	13
	23c	Discuss any limitations of the review processes used.	13
	23d	Discuss implications of the results for practice, policy, and future research.	14
**OTHER INFORMATION**			
Registration and protocol	24a	Provide registration information for the review, including register name and registration number, or state that the review was not registered.	4
	24b	Indicate where the review protocol can be accessed, or state that a protocol was not prepared.	4
	24c	Describe and explain any amendments to information provided at registration or in the protocol.	-
Support	25	Describe sources of financial or non-financial support for the review, and the role of the funders or sponsors in the review.	15
Competing interests	26	Declare any competing interests of review authors.	15
Availability of data, code and other materials	27	Report which of the following are publicly available and where they can be found: template data collection forms; data extracted from included studies; data used for all analyses; analytic code; any other materials used in the review.	7-9

From: Page MJ, McKenzie JE, Bossuyt PM, Boutron I, Hoffmann TC, Mulrow CD, et al. The PRISMA 2020 statement: an updated guideline for reporting systematic reviews. BMJ 2021;372:n71. doi: 10.1136/bmj.n71

S1. Prisma Checklist

S2. Search strategy for databases

1. Medline

Interface: Ovid MEDLINE(R) ALL<br><br>Date of Search: February 12, 2024<br><br>Number of hits: 681<br><br>Comment: In Ovid, two or more words are automatically searched as phrases; i.e. no quotation marks are needed	Field labels<br><br>• exp/ = exploded MeSH term<br>• / = non exploded MeSH term<br>• .ti,ab,kf. = title, abstract and author keywords<br>• adjx = within x words, regardless of order<br>• * = truncation of word for alternate endings

Database(s): **Ovid MEDLINE(R) ALL** 1946 to February 09, 2024
Search Strategy:

#	Searches	Results
1	exp Lactic Acid/	49121
2	Polymers/ or Polyesters/	125572
3	1 and 2	7090

4	New-Fill.nm.	73
5	("Gana V" or lactic acid polymer or new fill or new-fill or newfill or PLLA or poly-d l-lactic acid or poly l lactic acid or poly levo lactic acid or "poly (lactic acid)" or "poly (lactic acid) polymer" or poly-l-lactic acid or polylactate or polylactic acid or sculptra).ti,ab,kf.	13515
6	3 or 4 or 5	17320
7	Skin Aging/	10353
8	Rejuvenation/	4056
9	exp Face/	175921
10	Dermal Fillers/	1565
11	exp Cosmetic Techniques/	59672
12	((chin* or cheek* or eye* or face or facial or forehead or lip? or midface or midfacial or mouth or nasolabial or naso-labial or nose or perioral or skin*) adj4 (aesthetic* or age or aged or ageing or aging or atroph* or cosmetic* or cutaneous or dermal thickness or enhancement* or enhancing or esthetic* or fold? or laxity or photodamaged or photoaging or photoage* or regenerat* or rejuvenation* or restructur* or restor* or sagging or skin thickness or skin-tightening or soft-tissue augmentation or subcutaneous or tissue distribution or tissue response or tissue support or volum* or wrinkle* or wrinkling)).ti,ab,kf.	90487
13	(anti-wrinkle* or reduc* wrinkle*).ti,ab,kf.	453
14	((cosmetic* or aesthetic* or estethic*) adj3 (medicine or method* or techni*)).ti,ab,kf.	3501

15	(volume* adj3 (enhanc* or maint* or restor*)).ti,ab,kf.	8217
16	((cosmetic* or dermal or face or facial or injectable or injection* or skin) adj3 (filler* or filling)).ti,ab,kf.	3276
17	((cosmetic* or dermal or face or facial or skin or lactic acid polymer or new fill or new-fill or newfill or PLLA or poly-d l-lactic acid or poly l lactic acid or poly levo lactic acid or "poly (lactic acid)" or "poly (lactic acid) polymer" or poly-l-lactic acid or polylactate or polylactic acid or sculptra) adj3 (injection* or injectable)).ti,ab,kf.	3234
18	(atroph* scar* or acne scar* or lipoatroph*).ti,ab,kf.	3674
19	or/7-18	321300
20	6 and 19	681

2. Embase

Interface: embase.com<br><br>Date of Search: February 12, 2024<br><br>Number of hits: 1099<br><br>Comment: Emtree is the controlled vocabulary in Embase	Field labels<br><br>• /exp = exploded Emtree term<br>• /de = non exploded Emtree term<br>• ti,ab,kw = title, abstract and author keywords<br>• NEAR/x = within x words, regardless of order<br>• * = truncation of word for alternate endings

No.	Query	Results
#18	#3 AND #17	1099
#17	#4 OR #5 OR #6 OR #7 OR #8 OR #9 OR #10 OR #11 OR #12 OR #13 OR #14 OR #15 OR #16	317353
#16	'atroph* scar*':ti,ab,kw OR 'acne scar*':ti,ab,kw OR lipoatroph*:ti,ab,kw	5306
#15	((cosmetic* OR dermal OR face OR facial OR skin OR 'lactic acid polymer' OR 'new fill' OR 'new fill' OR newfill OR plla OR 'poly-d l-lactic acid' OR 'poly l lactic acid' OR 'poly levo lactic acid' OR 'poly (lactic acid)' OR 'poly (lactic acid) polymer' OR 'poly-l-lactic acid' OR polylactate OR 'polylactic acid' OR sculptra) NEAR/3 (injection* OR injectable)):ti,ab,kw	4248
#14	((cosmetic* OR dermal OR face OR facial OR injectable OR injection* OR skin) NEAR/3 (filler* OR filling)):ti,ab,kw	3824
#13	(volume* NEAR/3 (enhanc* OR maint* OR restor*)):ti,ab,kw	10825
#12	((cosmetic* OR aesthetic* OR estethic*) NEAR/3 (medicine OR method* OR techni*)):ti,ab,kw	5493
#11	'anti wrinkle*':ti,ab,kw OR 'reduc* wrinkle*':ti,ab,kw	611
#10	((chin* OR cheek* OR eye* OR face OR facial OR forehead OR lip$ OR midface OR midfacial OR mouth OR nasolabial OR 'naso labial' OR nose OR perioral OR skin*) NEAR/4 (aesthetic* OR age OR aged OR ageing OR aging OR atroph* OR cosmetic* OR cutaneous OR 'dermal thickness' OR	121102

	enhancement* OR enhancing OR esthetic* OR fold$ OR laxity OR photodamaged OR photoaging OR photoage* OR regenerat* OR rejuvenation* OR restructur* OR restor* OR sagging OR 'skin thickness' OR 'skin tightening' OR 'soft-tissue augmentation' OR subcutaneous OR 'tissue distribution' OR 'tissue response' OR 'tissue support' OR volum* OR wrinkle* OR wrinkling)):ti,ab,kw	
#9	'photoaging'/de OR 'skin regeneration'/de OR 'skin thickness'/de OR 'skin tightness'/de OR 'sagging skin'/de OR 'wrinkle'/de	12292
#8	'esthetic surgery'/exp OR 'esthetics'/de OR 'cosmetic dermatology'/de	40419
#7	'injectable dermal implant'/de	1288
#6	'face'/exp	145941
#5	'rejuvenation'/de	6694
#4	'cutaneous parameters'/de	10390
#3	#1 OR #2	19067
#2	'gana v':ti,ab,kw OR 'lactic acid polymer':ti,ab,kw OR 'new fill':ti,ab,kw OR newfill:ti,ab,kw OR plla:ti,ab,kw OR 'poly-d l-lactic acid':ti,ab,kw OR 'poly l lactic acid':ti,ab,kw OR 'poly levo lactic acid':ti,ab,kw OR 'poly (lactic acid)':ti,ab,kw OR 'poly (lactic acid) polymer':ti,ab,kw OR 'poly-l-lactic acid':ti,ab,kw OR polylactate:ti,ab,kw OR 'polylactic acid':ti,ab,kw OR sculptra:ti,ab,kw	14025
#1	'polylactic acid'/de	13783

3. Web of Science Core Collection

Interface: Clarivate Analytics<br><br>Editions = A&HCI , ESCI , SCI-EXPANDED , SSCI<br><br>Date of Search: February 12, 2024<br><br>Number of hits: 707	Field labels<br><br>• TS/Topic = title, abstract, author keywords and Keywords Plus<br>• NEAR/x = within x words, regardless of order<br>• * = truncation of word for alternate endings<br><br>Note: the *Exact search*-function was used for all the searches

#	Search Query	Results
1	TS=("Gana V" OR "lactic acid polymer" OR "new fill" OR new-fill OR newfill OR PLLA OR "poly-d l-lactic acid" OR "poly l lactic acid" OR "poly levo lactic acid" OR "poly (lactic acid)" OR "poly (lactic acid) polymer" OR "poly-l-lactic acid" OR polylactate OR "polylactic acid" OR sculptra)	35828
2	TS=((chin* OR cheek* OR eye* OR face OR facial OR forehead OR lip$ OR midface OR midfacial OR mouth OR nasolabial OR naso-labial OR nose OR perioral OR skin*) NEAR/4 (aesthetic* OR age OR aged OR ageing OR aging OR atroph* OR cosmetic* OR cutaneous OR "dermal thickness" OR enhancement* OR enhancing OR esthetic* OR fold$ OR laxity OR photodamaged OR photoaging OR photoage* OR regenerat* OR rejuvenation* OR restructur* OR restor* OR sagging OR "skin thickness" OR skin-tightening OR "soft-tissue augmentation" OR subcutaneous OR "tissue distribution" OR "tissue response" OR "tissue support" OR volum* OR wrinkle* OR wrinkling))	110653
3	TS=(anti-wrinkle* OR "reduc* wrinkle*")	631

4	TS=((cosmetic* OR aesthetic* OR estethic*) NEAR/3 (medicine OR method* OR techni*))	6403
5	TS=(volume* NEAR/3 (enhanc* OR maint* OR restor*))	13686
6	TS=((cosmetic* OR dermal OR face OR facial OR injectable OR injection* OR skin) NEAR/3 (filler* OR filling))	4251
7	TS=((cosmetic* OR dermal OR face OR facial OR skin OR "lactic acid polymer" OR "new fill" OR new-fill OR newfill OR PLLA OR "poly-d l-lactic acid" OR "poly l lactic acid" OR "poly levo lactic acid" OR "poly (lactic acid)" OR "poly (lactic acid) polymer" OR "poly-l-lactic acid" OR polylactate OR "polylactic acid" OR sculptra) NEAR/3 (injection* OR injectable))	4053
8	TS=("atroph* scar*" OR "acne scar*" OR lipoatroph*)	4105
9	#2 OR #3 OR #4 OR #5 OR #6 OR #7 OR #8	137920
10	#1 AND #9	707

4. Cinahl

Interface: Ebsco<br><br>Date of Search: February 12, 2024<br><br>Number of hits: 99	Field labels<br><br>• MH+ = exploded Cinahl Heading<br>• MH = non exploded Cinahl Heading<br>• TI = title<br>• AB = abstract<br>• Nx = within x words, regardless of order<br>• * = truncation of word for alternate endings<br><br>Note: sometimes “quotation marks” are needed for single search terms to avoid automatic term mapping (lemmatization)1

#	Query	Results
S19	S5 AND S18	99
S18	S6 OR S7 OR S8 OR S9 OR S10 OR S11 OR S12 OR S13 OR S14 OR S15 OR S16 OR S17	51,705
S17	TI (("atroph* scar*" or "acne scar*" or lipoatroph*)) OR AB (("atroph* scar*" or "acne scar*" or lipoatroph*))	666
S16	TI (((cosmetic* OR dermal OR face OR facial OR skin OR "lactic acid polymer" OR "new fill" OR new-fill OR newfill OR PLLA OR "poly-d l-lactic acid" OR "poly l lactic acid" OR "poly levo lactic acid" OR "poly (lactic acid)" OR "poly (lactic acid) polymer" OR "poly-l-lactic acid" OR polylactate OR "polylactic acid" OR sculptra) N3 (injection* OR injectable))) OR AB (((cosmetic* OR dermal OR face OR facial OR skin OR "lactic acid polymer" OR "new fill" OR new-fill OR newfill OR PLLA OR "poly-d l-lactic acid" OR "poly l lactic acid" OR "poly levo lactic acid" OR "poly (lactic acid)" OR "poly	677

	(lactic acid) polymer" OR "poly-l-lactic acid" OR polylactate OR "polylactic acid" OR sculptra) N3 (injection* OR injectable)))	
S15	TI (((cosmetic* OR dermal OR face OR facial OR injectable OR injection* OR skin) N3 (filler* OR filling))) OR AB (((cosmetic* OR dermal OR face OR facial OR injectable OR injection* OR skin) N3 (filler* OR filling)))	718
S14	TI ((volume* N3 (enhanc* OR maint* OR restor*))) OR AB ((volume* N3 (enhanc* OR maint* OR restor*)))	1,660
S13	TI (((cosmetic* OR aesthetic* OR estethic*) N3 (medicine OR method* OR techni*))) OR AB (((cosmetic* OR aesthetic* OR estethic*) N3 (medicine OR method* OR techni*)))	753
S12	TI ((anti-wrinkle* or "reduc* wrinkle*")) OR AB ((anti-wrinkle* or "reduc* wrinkle*"))	88
S11	TI (((chin* OR cheek* OR eye* OR face OR facial OR forehead OR lip# OR midface OR midfacial OR mouth OR nasolabial OR naso-labial OR nose OR perioral OR skin*) N4 (aesthetic* OR age OR aged OR ageing OR aging OR atroph* OR cosmetic* OR cutaneous OR "dermal thickness" OR enhancement* OR enhancing OR esthetic* OR fold# OR laxity OR photodamaged OR photoaging OR photoage* OR regenerat* OR rejuvenation* OR restructur* OR restor* OR sagging OR "skin thickness" OR skin-tightening OR "soft-tissue augmentation" OR subcutaneous OR "tissue distribution" OR "tissue response" OR "tissue support" OR volum* OR wrinkle* OR wrinkling))) OR AB (((chin* OR cheek* OR eye* OR face OR facial OR forehead OR lip# OR midface OR midfacial OR mouth OR nasolabial OR naso-labial OR nose OR perioral OR skin*) N4 (aesthetic* OR age OR aged OR ageing OR aging OR atroph* OR cosmetic* OR cutaneous OR "dermal thickness" OR enhancement* OR enhancing OR esthetic* OR	20,137

	fold# OR laxity OR photodamaged OR photoaging OR photoage* OR regenerat* OR rejuvenation* OR restructur* OR restor* OR sagging OR "skin thickness" OR skin-tightening OR "soft-tissue augmentation" OR subcutaneous OR "tissue distribution" OR "tissue response" OR "tissue support" OR volum* OR wrinkle* OR wrinkling)))	
S10	(MH "Cosmetic Techniques+")	5,027
S9	(MH "Dermal Fillers")	329
S8	(MH "Face+")	24,356
S7	(MH "Rejuvenation")	1,105
S6	(MH "Skin Aging")	2,549
S5	S3 OR S4	813
S4	TI (("Gana V" OR "lactic acid polymer" OR "new fill" OR new-fill OR newfill OR PLLA OR "poly-d l-lactic acid" OR "poly l lactic acid" OR "poly levo lactic acid" OR "poly (lactic acid)" OR "poly (lactic acid) polymer" OR "poly-l-lactic acid" OR polylactate OR "polylactic acid" OR sculptra)) OR AB (("Gana V" OR "lactic acid polymer" OR "new fill" OR new-fill OR newfill OR PLLA OR "poly-d l-lactic acid" OR "poly l lactic acid" OR "poly levo lactic acid" OR "poly (lactic acid)" OR "poly (lactic acid) polymer" OR "poly-l-lactic acid" OR polylactate OR "polylactic acid" OR sculptra))	525
S3	S1 AND S2	420
S2	(MH "Polymers+") OR (MH "Polyesters")	44,575
S1	(MH "Lactic Acid")	3,470
